# Clinical features and prognosis analysis of patients with follicular lymphoma: a real-world study in China

**DOI:** 10.1007/s00277-025-06617-2

**Published:** 2025-09-20

**Authors:** Yang Su, Qi Hu, Weili Zhao, Chi Liu, Li Wang, Shu Cheng, Pengpeng Xu, Linlin Liu, Wenzhong Yang

**Affiliations:** 1 Department of Hematology, Shanghai Municipal Hospital of Traditional Chinese Medicine, Shanghai University of Traditional Chinese Medicine, Shanghai, 200071 China; 2 Shanghai Institute of Hematology, State Key Laboratory of Medical Genomics, National Research Center for Translational Medicine at Shanghai, Ruijin Hospital, Shanghai Jiao Tong University School of Medicine, Shanghai, 200025 China; 3 Department of Geriatrics Center & National Clinical Research Center for Aging and Medicine, Jing’an District Centre Hospital of Shanghai, Fudan University, Shanghai, 200040 China; 4 Department of Cardiology, Shanghai Seventh People’s Hospital, Shanghai University of Traditional Chinese Medicine, Shanghai, 200137 China; 5 Department of Hematology, Shanghai Punan Hospital of Pudong New District, Shanghai, 200125 China

**Keywords:** Follicular Lymphoma, Prognosis, Clinical features, Pathological grade, NK cells

## Abstract

Follicular lymphoma (FL) is a common indolent B-cell lymphoma with heterogeneous clinical behavior. Prognostic data from Chinese real-world populations remain Limited. We retrospectively analyzed 118 patients with newly diagnosed FL treated at our center from January 2022 to December 2024. Clinical features, laboratory indicators, immune markers, and treatment responses were evaluated. Survival outcomes were analyzed using Kaplan–Meier and Cox regression methods. The follow-up ended on January 31, 2025, with a median follow-up duration of 11.2 months (range, 1.1–36.0 months). The 2-year overall survival (OS) and progression-free survival (PFS) rates were 90.1% and 69.7%, respectively. The overall complete response (CR) rate was 60.2%. In univariate analysis, several factors including pathological grade 3B/composite histology, B symptoms, low NK cell percentage (< 8%), low CD4^+^ T cell levels (< 30%), CD5 positivity, CD10 negativity, and MUM1 positivity were significantly associated with poor OS and/or PFS. Multivariate Cox regression revealed that pathological grade 3B/composite histology was an independent risk factor for PFS (HR 2.933, *P* = 0.035), while NK cell percentage < 8% independently predicted worse OS (HR 0.067, *P* = 0.005). This study validates the prognostic significance of pathological grade and NK cell levels in a Chinese real-world FL population. Peripheral blood NK cell percentage may serve as a clinically useful biomarker for host immune status and prognosis. The findings support further investigation into immune-based prognostic markers and therapeutic strategies in FL.

## Introduction

Follicular lymphoma (FL) is the malignant counterparts of normal germinal center B-cells. FL is the second most common lymphoma diagnosed in the United States and Western Europe, accounting for approximately 35% of all non-Hodgkin lymphomas (NHLs), and 70% of indolent lymphomas [1, 2]. FL is classified into three distinct pathologic grades (i.e., FL1-3) according to the number of centroblasts per high-power field [2]. FL3 can further be subdivided into FL3a and FL3b in the current WHO classification. FL3b is clinically and biologically more like diffuse large B cell lymphoma and is treated as such.

As an indolent, often asymptomatic yet incurable disease, FL helped define observation, “watch and wait”, as an acceptable, in fact standard, approach and had a long natural course, so patients with FL frequently require multiple treatments during their disease course [3]. While FL remains incurable, overall survival (OS) continues to improve due to improvements in diagnostic tools and supportive care, the development of the monoclonal anti-CD20 antibody rituximab, obinutuzumab, and the increasing number of FDA-approved therapies[4–6]. The prognosis of follicular lymphoma was significantly improved, with the median overall survival time of 20 years and the 5-year overall survival rates reaching 90% [4]. Despite the improved effectiveness of chemoimmunotherapy regimens, approximately 20% of patients with FL experience progression of disease (POD) within 2 years of first-line therapy [7]. However, the frequency of transformation from FL to aggressive non-Hodgkin’s lymphoma varies between from 10 to 70%, and the annual risk of transformation of FL is 3%. Despite intensification of therapy, transformation of FL to aggressive lymphoma typically results in poor outcome with the majority of patients dying within 1 year [8]. Most of the above evidence comes from foreign clinical studies, and there are few clinical studies related to follicular lymphoma in China. Due to FL with obvious heterogeneity on ethnicity and region [9], more clinical studies on Chinese FL patients are needed to guide clinical practice.

Accordingly, we conducted a retrospective study to explore the clinical features and prognostic factors of Chinese patients with FL in the real word to provide real data for clinical and scientific research.

## Material and methods

### Patients and treatments

A total of 118 FL patients (53 females, 65 males; median age, 63 year; range, 33–84) were retrospectively screened and reviewed for diagnostic conformation at Shanghai Municipal Hospital of Traditional Chinese Medicine Affiliated of Shanghai University of Traditional Chinese Medicine and Rui Jin Hospital Affiliated of Shanghai Jiao Tong University School of Medicine from January 2022 to December 2024, and they all met the WHO classification criteria for lymphoid tissue tumors. All cell types (small-cell, mixed, or large-cell FL) could be included in the study. Patients who met the following criteria were included: (1) patients with newly diagnosed stage II to IV FL; (2) patients with histologic grade 1 to 3b confirmed by lymph node biopsy; (3) patients who received the first line standard R-CHOP (n = 38) (Rituximab 375 mg/m^2^ on Day 1; Cyclophosphamide 750 mg/m^2^ on Day 1; Doxorubicin 50 mg/m^2^ on Day 1; Vincristine 1.4 mg/m^2^ on Day 1; Prednisone 100 mg/m^2^ on Days 1–5; repeat every 21 days), GB (n = 51) (Ofatumumab 1000 mg on Day 1,8,15 of the first cycle, followed by 1000 mg on Day 1; Bendamustine 90 mg/m^2^ on Days 1–2; repeated every 28 days), BR (n = 14) (Rituximab 375 mg/m^2^ on Day 1; Bendamustine 90 mg/m^2^ on Days 1–2; repeated every 28 days), R2 (n = 11) (Rituximab 375 mg/m^2^ on Day 1; Lenalidomide 10-25 mg on Days 1–10; repeated every 28 days) or GR (n = 4) (Ofatumumab 1000 mg on Day 1,8,15 of the first cycle, followed by 1000 mg on Day 1; Lenalidomide 10-25 mg on Days 1–10; repeated every 28 days) chemoimmunotherapy in our center. We excluded patients who were < 18 years old, patients who lacked adequate clinical information, or those who were lost to follow-up. The clinical data are complete. Staging procedures included at least CT scans of the chest and whole abdomen, superficial lymph node ultrasound, or positron-emission tomography CT (PET-CT), bone marrow biopsy, routine blood counts, and biochemical examinations. Response was evaluated after induction, and definitions of complete response (CR), partial response (PR), stable disease (SD), and progressive disease (PD) were the standard [10]. The study was conducted in accordance with the Declaration of Helsinki and approved by the Hospital Clinic Institutional Review Board.

### Data collection and flow cytometry

Data collection includes detailed baseline demographics, pathological and clinical information, treatment and outcomes. Specially, we collected information on the following variables: age, involved lymph nodal sites, primary site, Ann Arbor stage, B symptom (defined as recurrent fever > 38.3℃, night sweats or the loss of > 10% body weight within 6 months), the risk of PRIMA-PI, FLIPI-2 score, hemoglobin level, albumin level, lactate dehydrogenase (LDH) level, β_2_-microglobulin (β_2-_MG) level, pathological grade, Ki-67 proliferation index, CD4^+^ T cells, CD8^+^T cells, NK cells, and the positivity for Bcl-2, Bcl-6, CD5, CD10, CD20,CD21 and MUM1.

The percentages of CD4^+^ T cells, CD8^+^ T cells, and NK cells in peripheral blood were measured using flow cytometry. Immunophenotyping of peripheral blood lymphocytes was analyzed by 18-colour flow cytometry (LSRFortessa & trade; BD Biosciences, USA) following standard operating procedures. The Ki-67 proliferation index and the expression of Bcl-2, Bcl-6, CD5, CD10, CD20, CD21, and MUM1 were assessed by immunohistochemistry (IHC) on paraffin-embedded lymph node biopsy specimens, and reviewed independently by hematopathologists.

### Outcomes of interest

The outcomes of interest were PFS and OS. PFS was defined as the time between initial treatment and relapse, death or the last follow-up. OS was defined as the time from initial treatment to death of any cause or the last follow-up. The last follow-up was January 31, 2025.

### Statistical analysis

Statistical analyses were performed in SPSS version 23 (SPSS, Inc., Chicago, IL, USA). Survival curves were plotted using the Kaplan–Meier method and compared using log-rank tests. Univariable log-rank test was applied to compare the prognostic value for PFS and OS. Variables with a p-value < 0.10 in the univariable analysis were considered candidates for inclusion in the multivariable Cox proportional hazards regression model. To reduce the risk of model overfitting given the sample size (n = 118), we Limited the final number of covariates to no more than 10. Variable selection was based on a combination of statistical significance, clinical relevance, and absence of multicollinearity. The multivariate model was further adjusted for treatment group as a potential confounder, and results were reported as hazard ratios (HRs) with corresponding 95% confidence intervals (CIs). All statistical tests were two-sided, and a p-value < 0.05 was considered statistically significant.

The receiver operating characteristic (ROC) curve was used to determine the 8% cut off of NK cells level and the 30% cut off of CD4^+^T cells level, as well as comparing different biomarkers for OS and PFS with Youden index. Immune cell levels were then classified using cut off values [11]. All tests were two-tailed and a P value < 0.05 was considered statistically significant.

## Results

### Baseline characteristics

A total of 118 patients meeting the criteria were included in the final analysis. Baseline characteristics of 118 FL patients are presented in Table 1. The median age was 63 (range, 33–84) years, and 85 (72%) patients were aged ≥ 60 years. There were 65 (55.1%) male patients, 42 (35.6%) had B symptom. There were 55 (46.6%) patients with bone marrow involvement, 93 (78.8%) patients with Ann Arbor stage III-IV, 40 (33.9%) patients with grade 3B and composite, 61 (51.7%) patients with FLIPI-2 score ≥ 3, 71 (60.2%) patients with the high risk of PRIMA-PI. 47 (39.8%) of FL patients had extranodal disease involvement ≥ 2. For laboratory data, 59 (50%) patients had hemoglobin (HB) < 120 g/L, 70 (59.3%) patients had albumin (ALB) < 40 g/L, 58 (49.2%) patients had elevated lactate dehydrogenase (LDH), and 62 (52.5%) patients had elevated β_2_-MG. Additionally, 62 (52.5%) patients had lower CD4^+^T cells levels (< 30%), 56 (47.5%) patients had lower NK cells levels (< 8%), and 82 (69.5%) patients had higher Ki-67 levels (≥ 30%). In terms of immunostaining, the probability of cases were positive for Bcl-2 (94.1%), Bcl-6 (96.6%), CD5 (48.3%), CD21 (96.6%), CD10 (57.6%), MUM1 (55.1%) and CD20 (100%).

**Table 1:** Baseline characteristics of the patients with FL (*N* = 118)

Characteristic	*n* (%)
Age (year), median (range)	
< 60	33
≥ 60	85
Gender	
Female	53
Male	65
Extranodal involvement ≥ 2	
Yes	47
No	71
B symptoms	
Yes	42
No	76
Bone marrow involvement	
Yes	55
No	63
Ann Arbor stage	
I/II	25
III/IV	93
PRIMA-PI	
low and intermediate-risk group	47
high-risk group	71
Histological grade	
1-3A	78
3B + composite	40
FLIPI-2	
< 3	57
≥ 3	61
HB (g/L) < 120	
Yes	59
No	59
ALB (g/L) < 40	
Yes	70
No	48
LDH (U/L) > UNL	
Yes	58
No	60
b2-MG (mg/L) > UNL	
Yes	62
No	56
CD4 + T cells	
< 30%	62
≥30%	56
NK cells	
< 8%	56
≥8%	62
Ki-67	
< 30%	36
≥30%	82
Bcl-2	
positive	111
negative	7
Bcl-6	
positive	114
negative	4
CD5	
positive	57
negative	61
CD21	
positive	114
negative	4
CD10	
Positive	68
negative	50
CD20	
positive	118
negative	0
MUM1	
positive	65
negative	53

### Survival outcomes by baseline characteristics

**Fig. 1:**
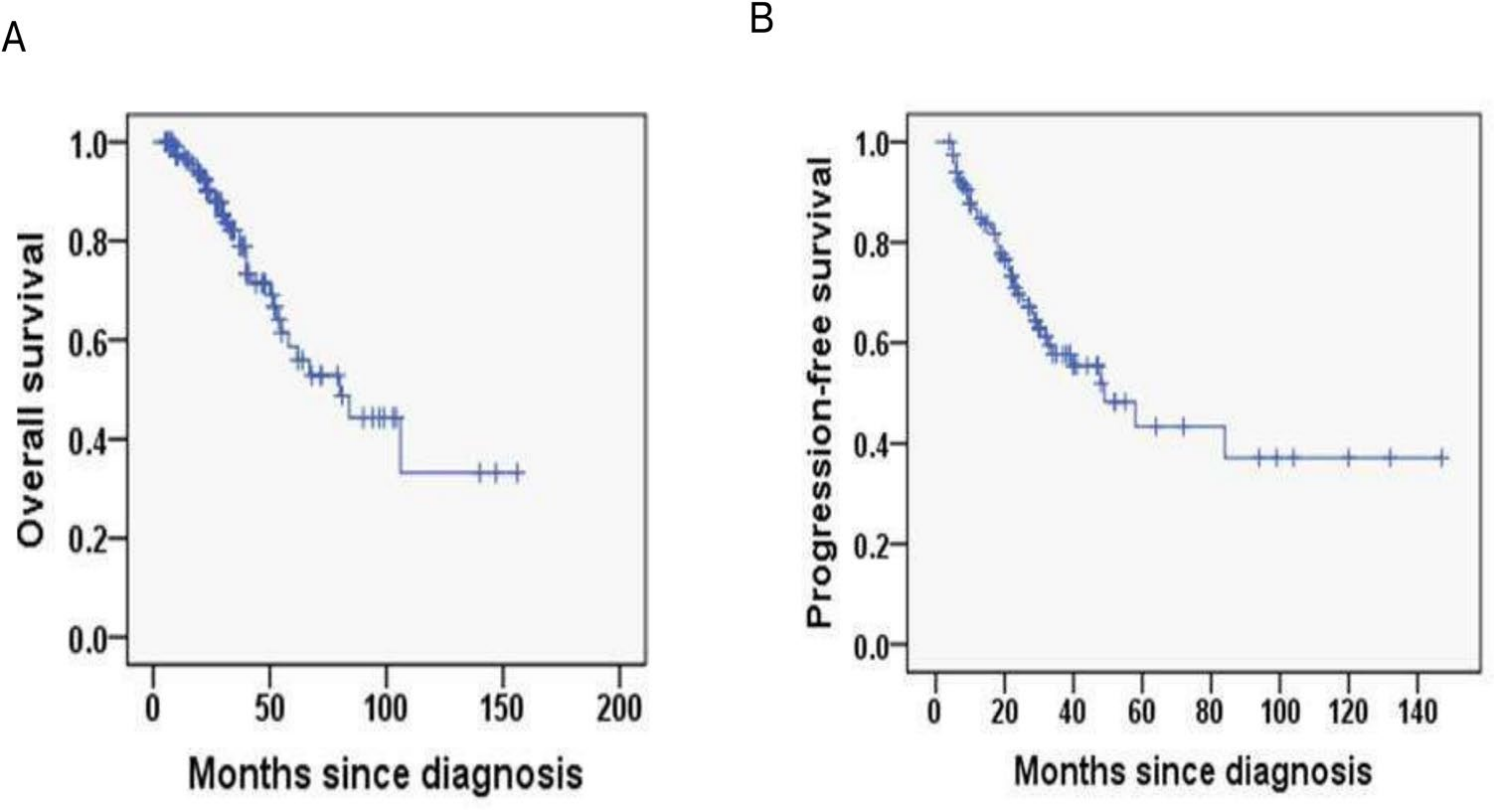
Overall survival (**A**) and progression-free survival (**B**) from time of diagnosis for 118 follicular lymphoma patients

**Fig. 2:**
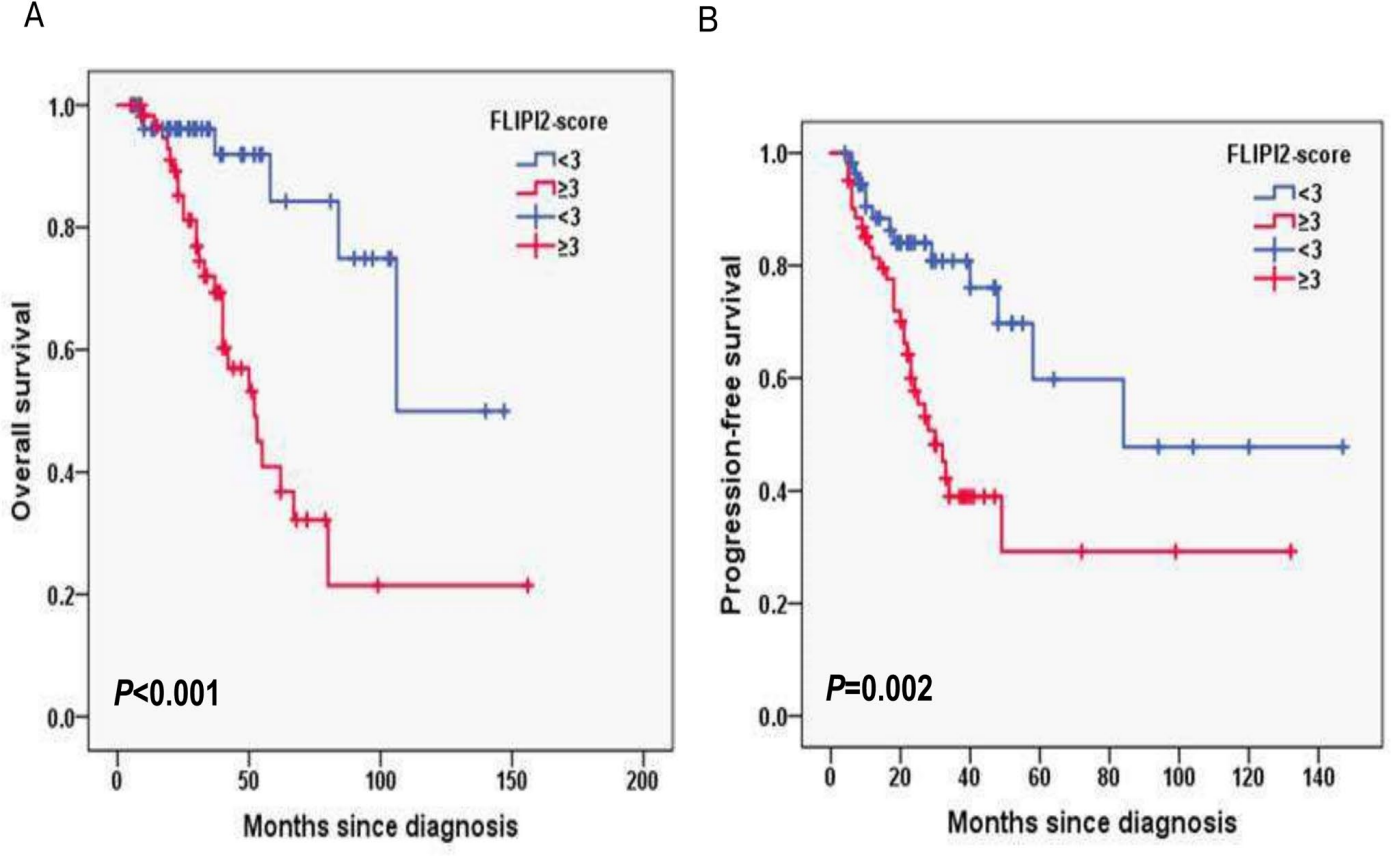
Overall survival (**A**) and progression-free survival (**B**) according to FLIPI-2 score (log-rank test FLIPI-2 < 3 vs. FLIPI-2 ≥ 3) in follicular lymphoma patients

**Fig. 3:**
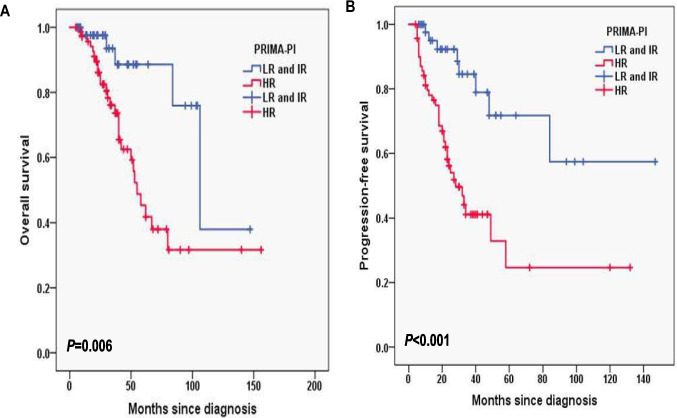
Overall survival (**A**) and progression-free survival (**B**) according to PRIMA-PI (log-rank test the low and intermediate-risk (LR, IR) group vs. high risk (HR) group) in follicular lymphoma patients

**Fig. 4:**
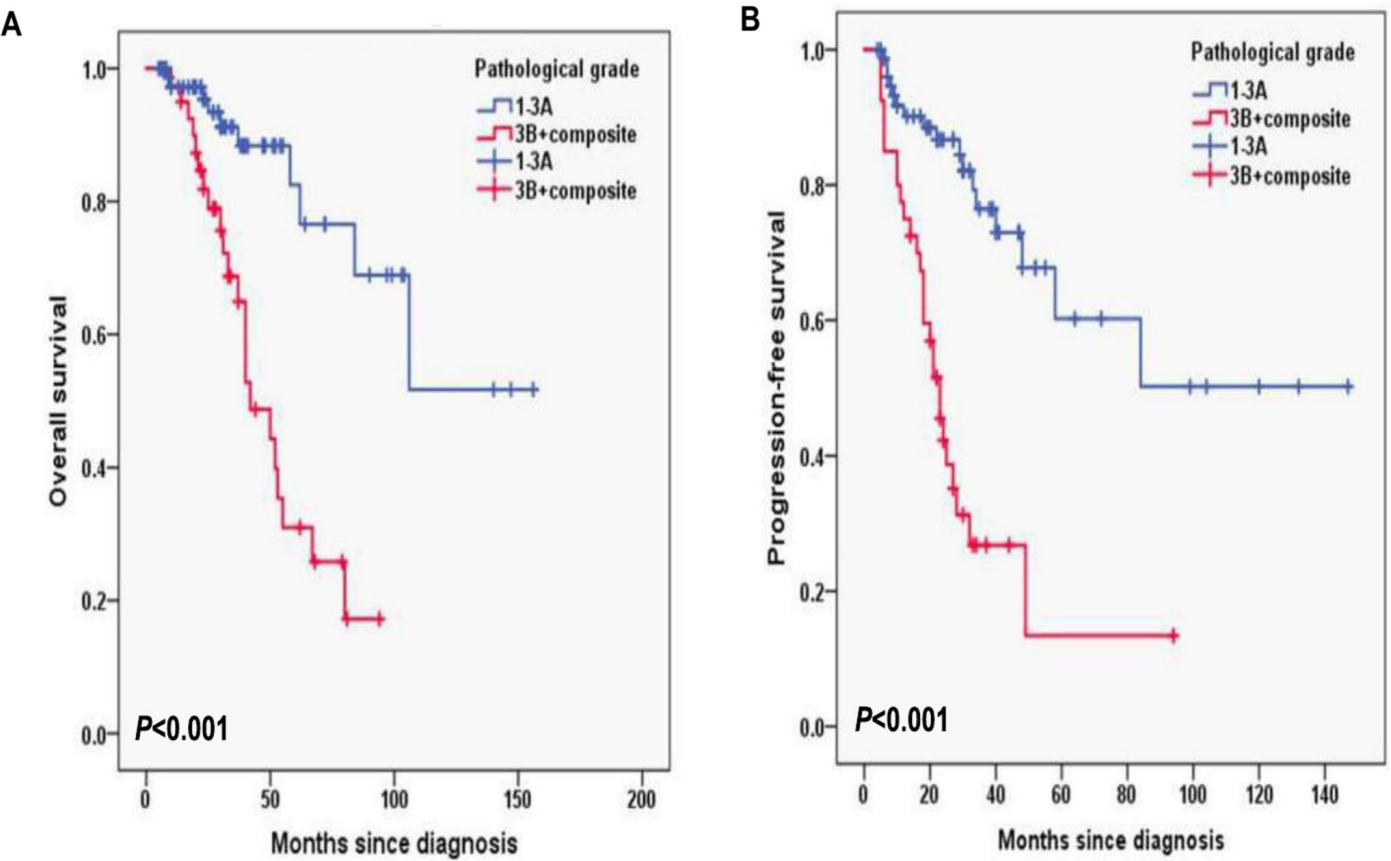
Overall survival (**A**) and progression-free survival (**B**) according to the pathological grade (log-rank test grade1-3A group vs. grade 3B, composite group) in follicular lymphoma patients

**Fig. 5:**
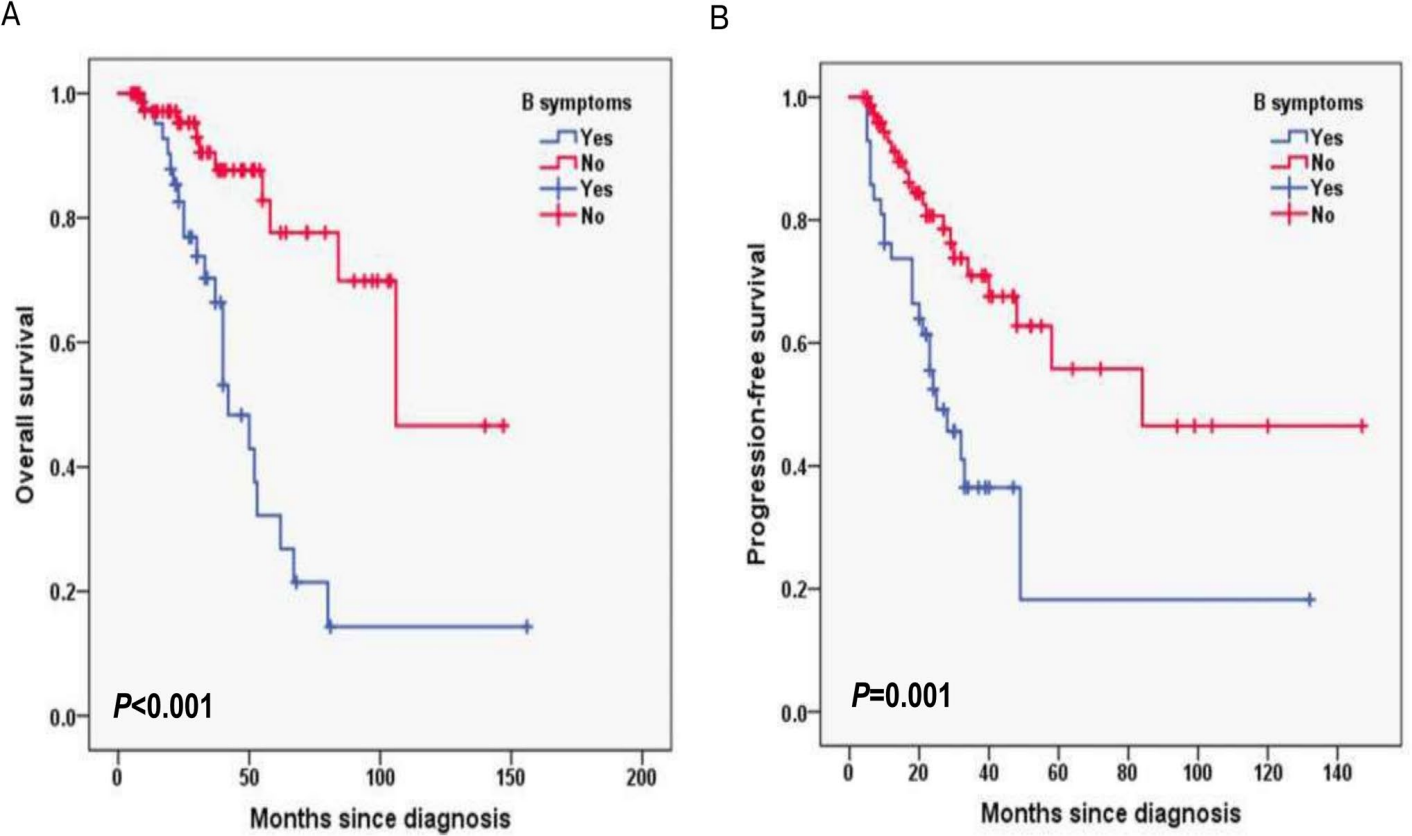
Overall survival (**A**) and progression-free survival (**B**) according to the B symptoms (log-rank test with B symptoms group vs. not with B symptoms group) in follicular lymphoma patients

In this cohort, the median follow-up duration of 11.2 months with interquartile range (IQR) (8.4–14.0). The 2-year OS rate was 90.1%, and the 2-year PFS rate was 69.7% (Fig. 1A, B). In FLIPI-2, the 3-year OS rates for the score < 3 and score ≥ 3 group were 96.1% and 72%, respectively (*P* < 0.001; Fig. 2A) and the 3-year PFS rates for the score < 3 and score ≥ 3 group were 80.8% and 39%, respectively (*P* = 0.002; Fig. 2B). In PRIMA-PI, the 3-year OS rates for the low and intermediate-risk (LR, IR) group and high-risk (HR) group were 93.5% and 76.1%, respectively (*P* = 0.006; Fig. 3A) and 3-year PFS rates for the LR, IR group and HR group were 84.6% and 41.1%, respectively (*P* < 0.001; Fig. 3B). In pathological grade, the 3-year OS rates for the grade1-3A group and grade 3B, composite group were 91.2% and 68.7%, respectively (*P* < 0.001; Fig. 4A) and 3-year PFS rates for the grade1-3A group and grade 3B, composite group were 76.5% and 26.8% (*P* < 0.001; Fig. 4B). In B symptoms, the 3-year OS rates for the patients with B symptoms group and not with B symptoms group were 70.3% and 90.5%, respectively (*P* < 0.001; Fig. 5A) and 3-year PFS rates for the patients with B symptoms group and not with B symptoms group were 36.5% and 70.9%, respectively (*P* = 0.001; Fig. 5B).

**Fig. 6:**
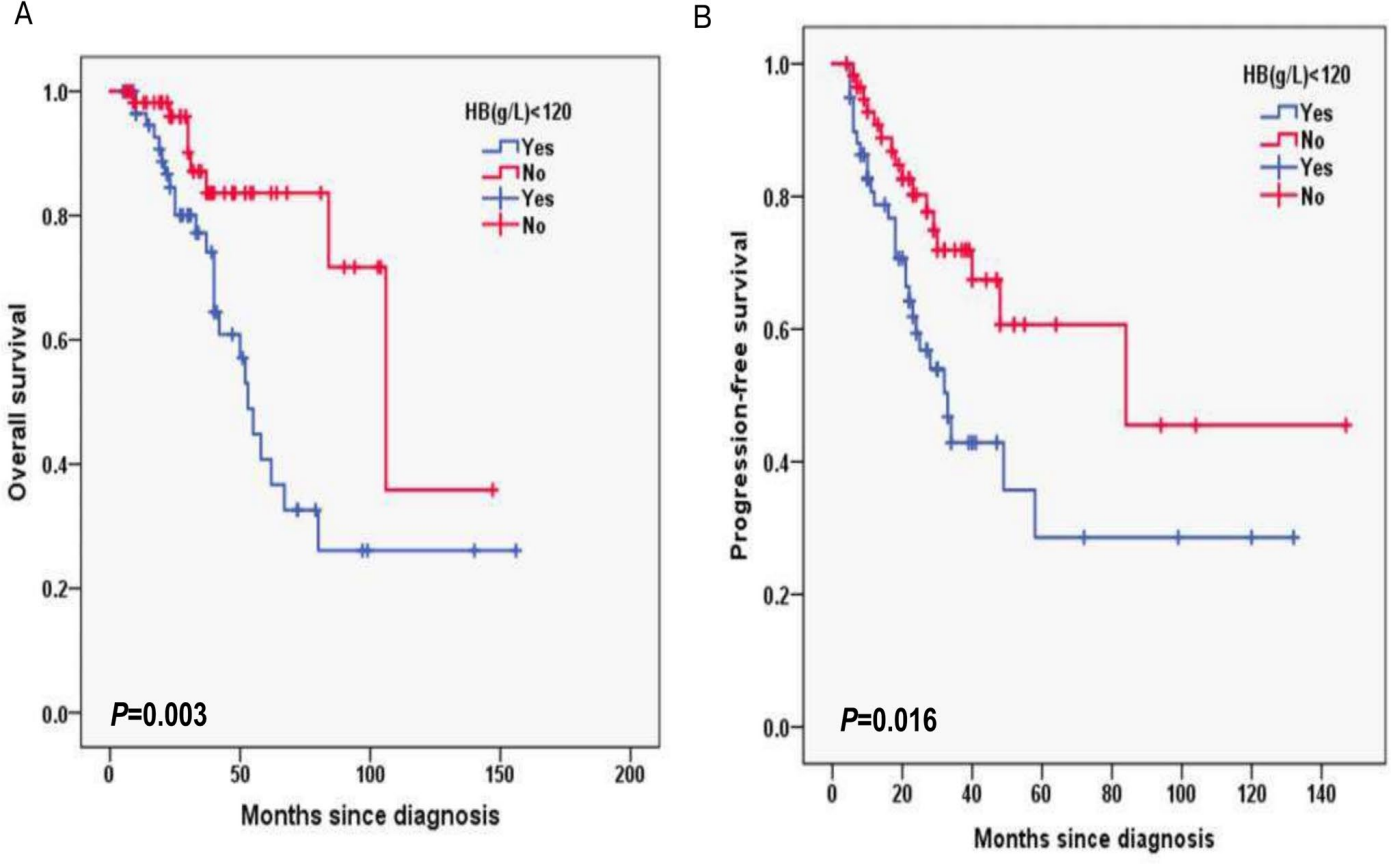
Overall survival (**A**) and progression-free survival (**B**) according to the level of hemoglobin (HB) (log-rank test HB < 120 g/L group vs. ≥ 120 g/L group) in follicular lymphoma patients

**Fig. 7:**
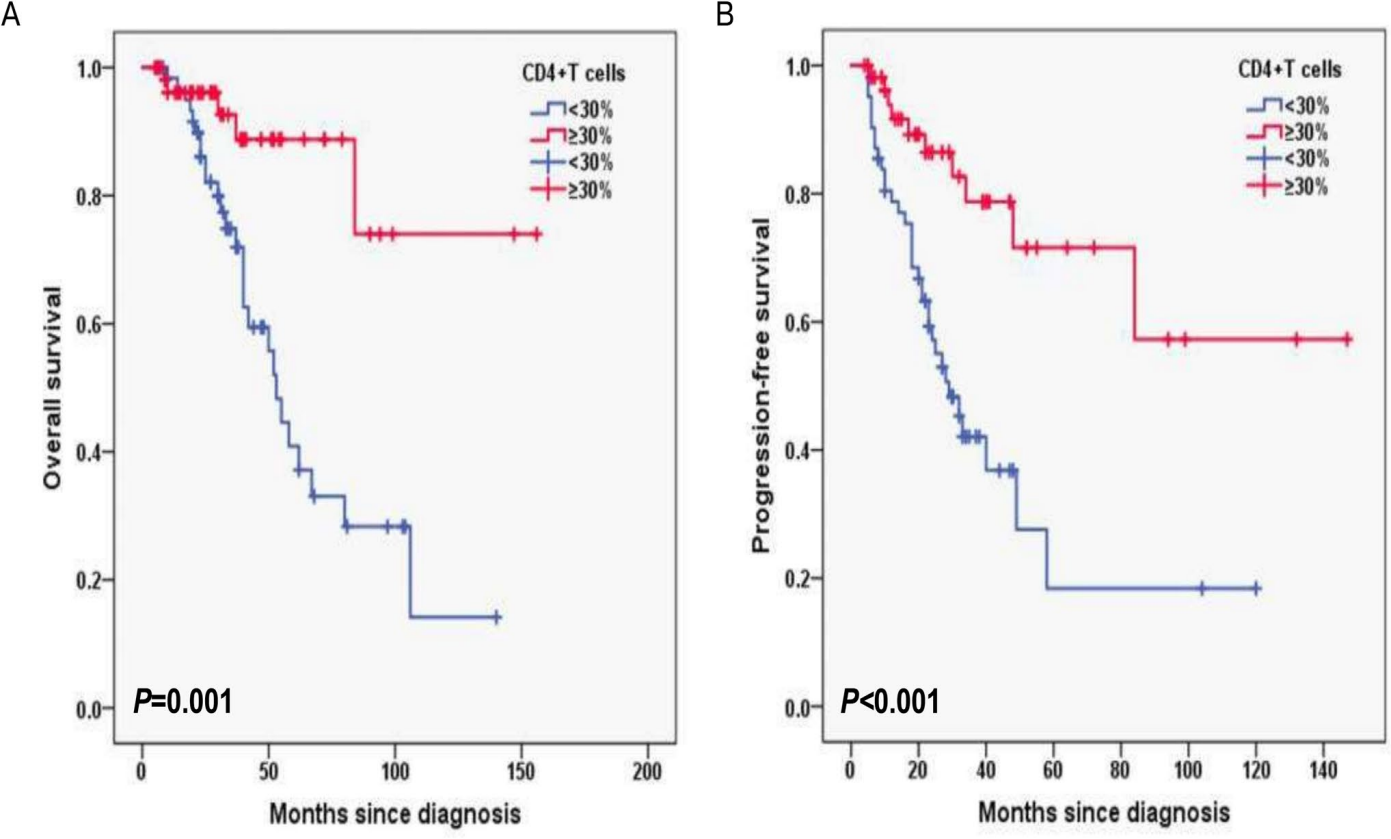
Overall survival (**A**) and progression-free survival (**B**) according to CD4 + T cells (log-rank test CD4 + T cells levels < 30% group vs. ≥ 30% group) in follicular lymphoma patients

**Fig. 8:**
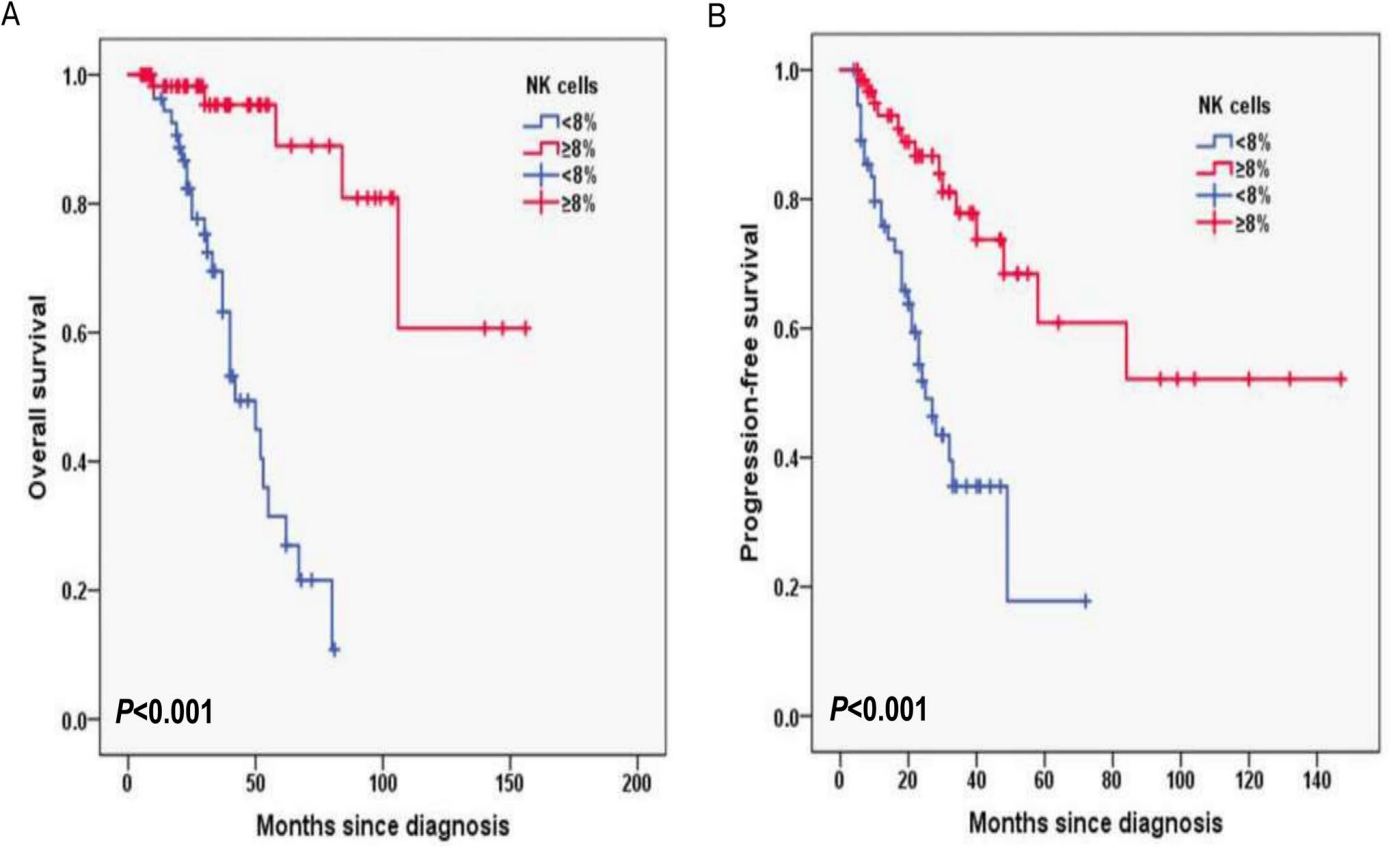
Overall survival (**A**) and progression-free survival (**B**) according to NK cells (log-rank test NK cells levels < 8% group vs. ≥ 8% group) in follicular lymphoma patients

**Fig. 9:**
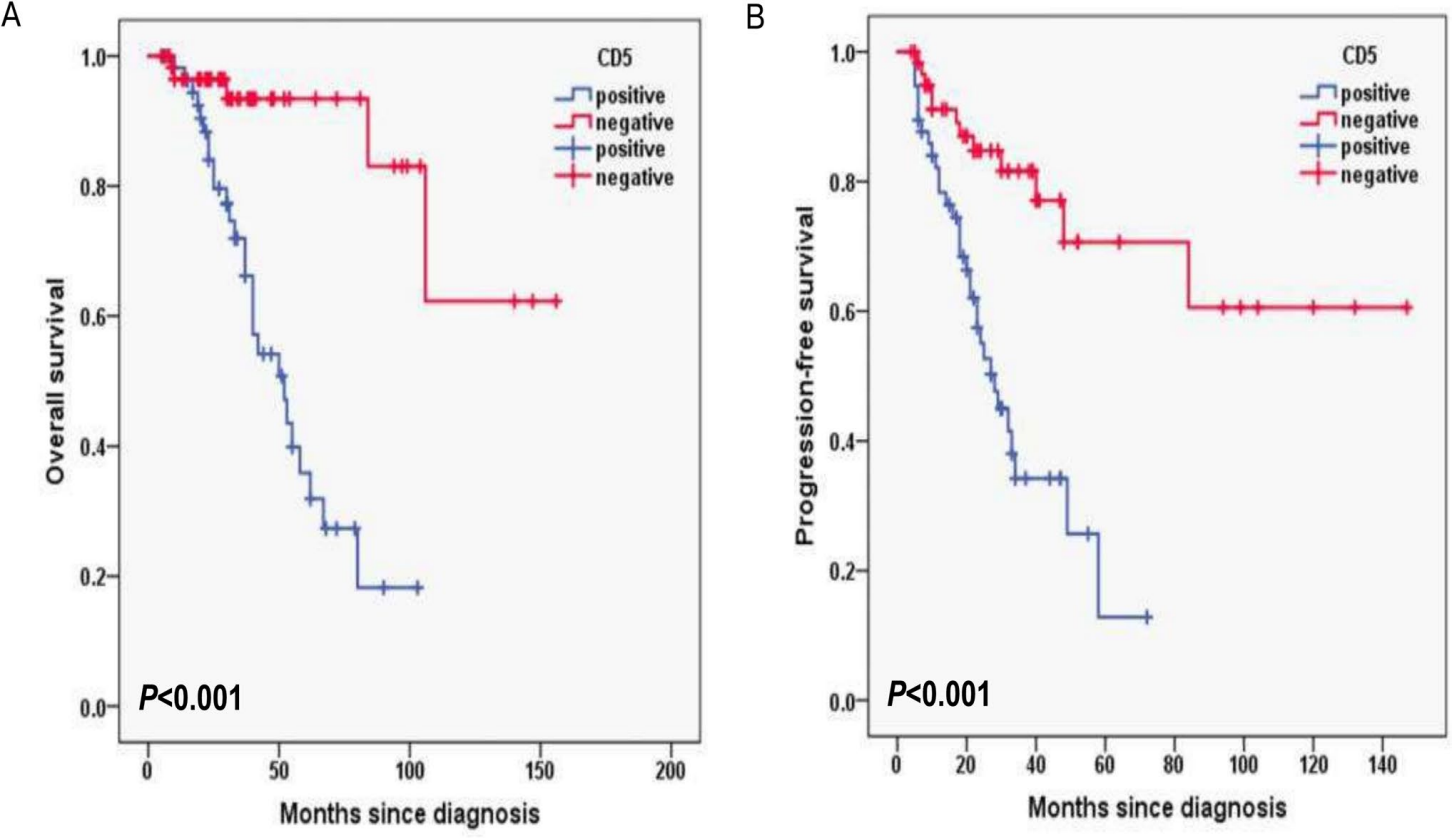
Overall survival (**A**) and progression-free survival (**B**) according to CD5 (log-rank test the positive group vs. negative group) in follicular lymphoma patients

**Fig. 10:**
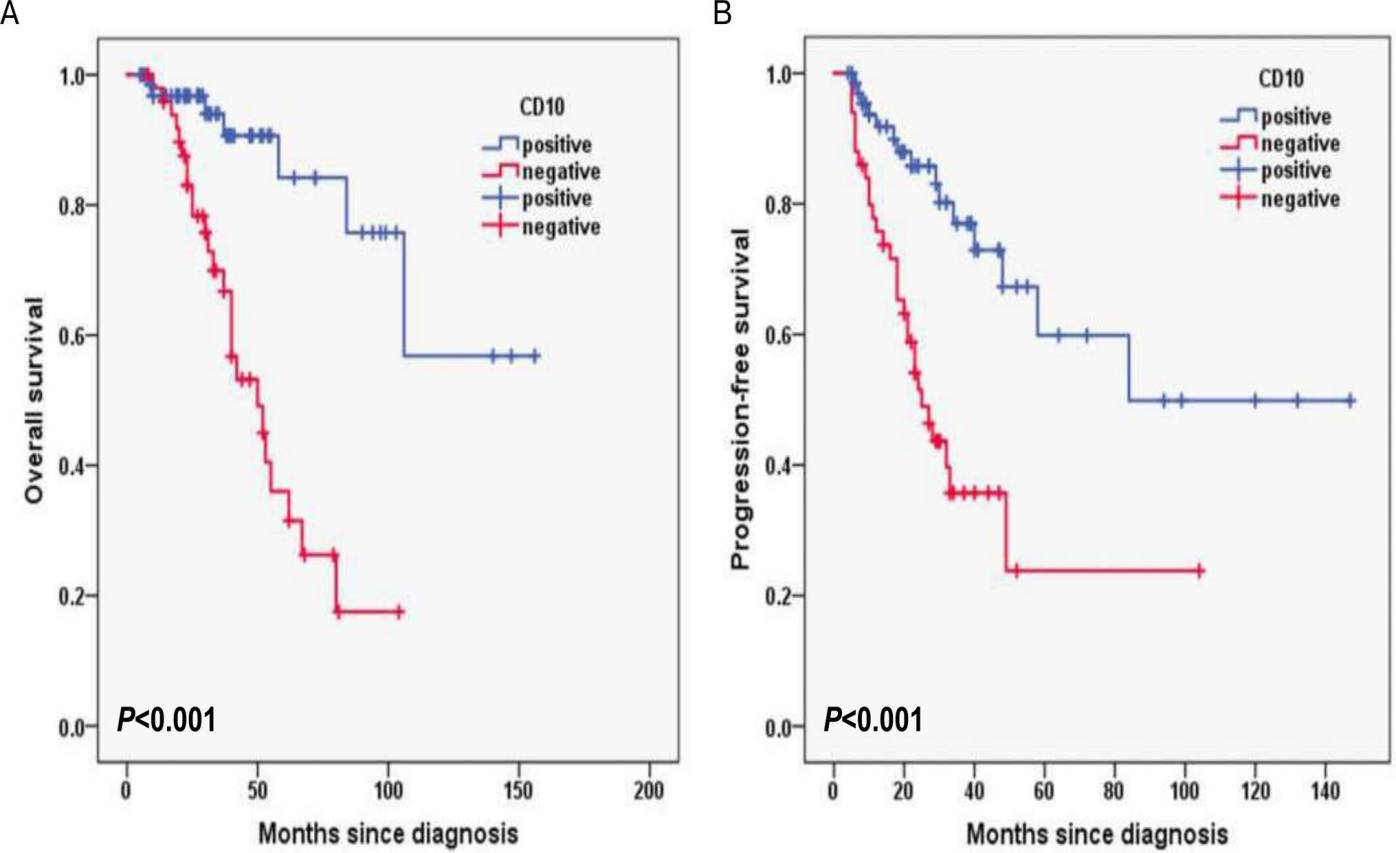
Overall survival (**A**) and progression-free survival (**B**) according to CD10 (log-rank test the positive group vs. negative group) in follicular lymphoma patients

**Fig. 11:**
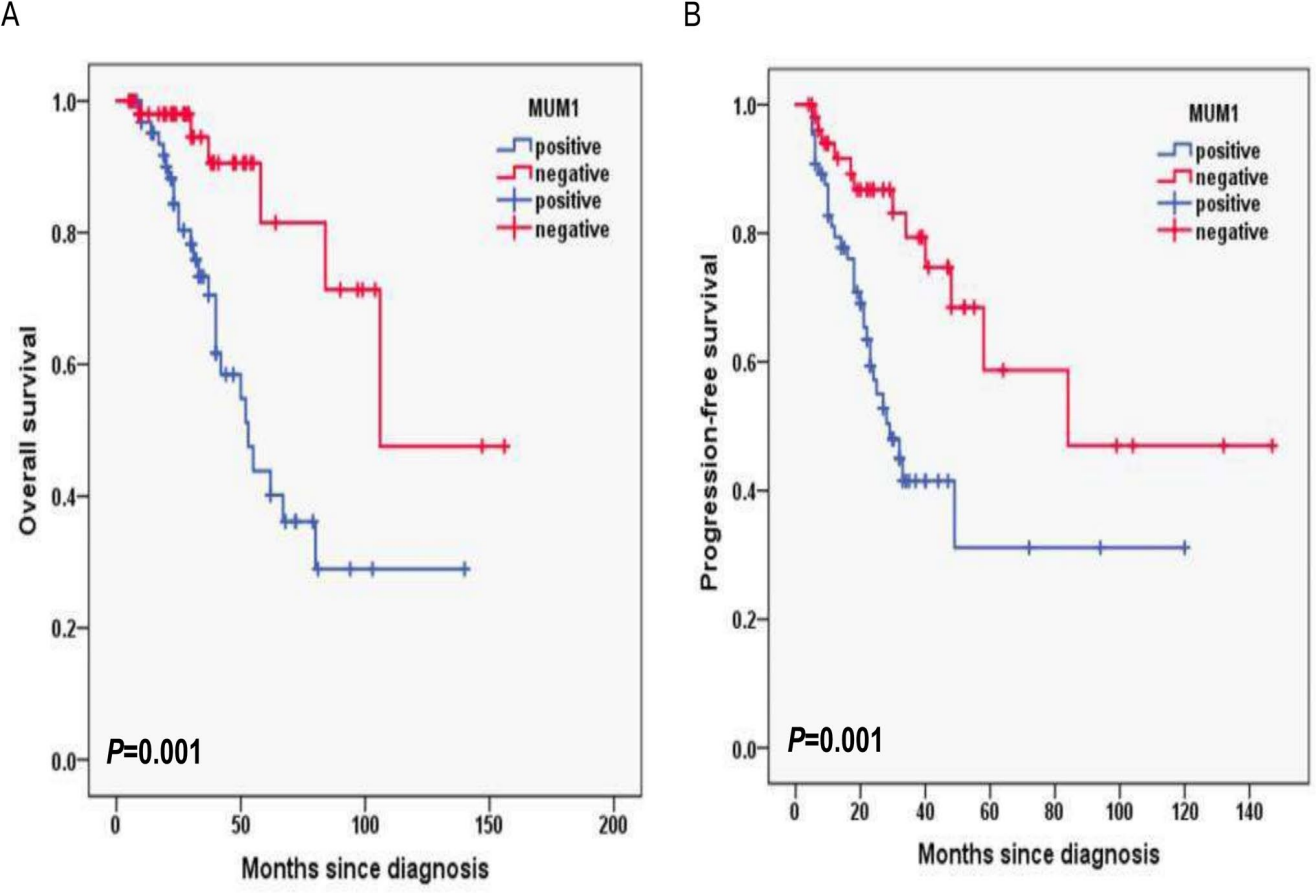
Overall survival (**A**) and progression-free survival (**B**) according to MUM1 (log-rank test the positive group vs. negative group) in follicular lymphoma patients

Additionally, for laboratory data, in HB, the 3-year OS rates for the HB < 120 g/L group and ≥ 120 g/L group were 77.2% and 87.1%, respectively (*P* = 0.003; Fig. 6A) and 3-year PFS rates for the HB < 120 g/L group and ≥ 120 g/L group were 42.9% and 71.9%, respectively (*P* = 0.016; Fig. 6B). In CD4^+^T cells, the 3-year OS rates for the CD4^+^T cells levels < 30% group and ≥ 30% group were 74.8% and 92.6%, respectively (*P* = 0.001; Fig. 7A) and 3-year PFS rates for the CD4^+^T cells levels < 30% group and ≥ 30% group were 42.1% and 78.7%, respectively (*P* < 0.001; Fig. 7B). In NK cells, the 3-year OS rates for the NK cells levels < 8% group and ≥ 8% group were 69.5% and 95.3%, respectively (*P* < 0.001; Fig. 8A) and 3-year PFS rates for the NK cells levels < 8% group and ≥ 8% group were 35.6% and 77.8%, respectively (*P* < 0.001; Fig. 8B). In terms of immunostaining, in CD5, the 3-year OS rates for positive group and negative group were 71.9% and 93.4%, respectively (*P* < 0.001; Fig. 9A) and 3-year PFS rates for positive group and negative group were 34.3% and 81.6%, respectively (*P* < 0.001; Fig. 9B). In CD10, the 3-year OS rates for positive group and negative group were 94% and 69.9%, respectively (*P* < 0.001; Fig. 10A) and 3-year PFS rates for positive group and negative group were 77% and 35.7%, respectively (*P* < 0.001; Fig. 10B). In MUM1, the 3-year OS rates for positive group and negative group were 73.3% and 94.5%, respectively (*P* = 0.001; Fig. 11A) and 3-year PFS rates for positive group and negative group were 41.5% and 79.4%, respectively (*P* = 0.001; Fig. 11B).

### Prognosis analysis of FL patients

Univariable log-rank test was applied to compare the prognostic value for PFS and OS. Pathological grade 3B and composite FL, with B symptoms, high risk of PRIMA-PI, FLIPI-2 score ≥ 3, hemoglobin level < 120 g/L, albumin level < 40 g/L, elevated serum LDH and β_2-_MG level, CD4^+^T cells < 30%, NK cells < 8%, Ki-67 proliferation index ≥ 30%, CD5 positive, CD10 negative and MUM1 positive were found to predict PFS and OS upon univariate analysis (*P* < 0.05) (Table 2).

**Table 2:** Overall survival and progression-free survival in univariate analysis in follicular lymphoma

Influencing factors	OS rate in 3 years with follow-up (%)	*P*	PFS rate in 3 years with follow-up (%)	*P*
B symptoms		< 0.001		< 0.001
Yes	91.2%		76.5%	
No	68.7%		26.8%	
Pathological grade		< 0.001		0.001
1-3A	70.3%		36.5%	
3B + composite	90.5%		70.9%	
PRIMA-PI		0.006		< 0.001
LR and IR	93.5%		84.6%	
HR	76.1%		41.1%	
FLIPI-2		< 0.001		0.002
< 3	96.1%		80.8%	
≥3	72.0%		39.0%	
HB		0.003		0.016
< 120 g/L	77.2%		42.9%	
≥120 g/L	87.1%		71.9%	
ALB		< 0.001		0.002
< 40 g/L	74.2%		43.9%	
≥40 g/L	97.7%		83.3%	
LDH		0.049		0.003
Normal	89.7%		72.3%	
Higher	75.5%		44.7%	
b_2_-MG		0.038		0.031
Normal	89.4%		69.2%	
Higher	76.3%		47.5%	
CD4 + T cells		0.001		< 0.001
< 30%	74.8%		42.1%	
≥30%	92.6%		78.7%	
NK cells		< 0.001		< 0.001
< 8%	69.5%		35.6%	
≥8%	95.3%		77.8%	
Ki-67		0.005		0.001
< 30%	91.5%		87.6%	
≥30%	78.9%		46.2%	
CD5		< 0.001		< 0.001
positive	71.9%		34.3%	
negative	93.4%		81.6%	
CD10		< 0.001		< 0.001
positive	94.0%		77.0%	
negative	69.9%		35.7%	
MUM1		0.001		0.001
positive	73.3%		41.5%	
negative	94.5%		79.4%	

Among the variables significantly associated with PFS or OS in the univariate analysis (*P* < 0.10), we selected a maximum of 10 covariates to include in the multivariate Cox regression model, based on both clinical relevance and statistical significance, while avoiding multicollinearity. These variables underwent confirmatory multivariate Cox regression analysis to identify independent prognostic factors (Table 3). The pathological grade was found to predict PFS (*P* = 0.035; HR 2.933; 95% CI 1.080–7.963), but not OS (*P* = 0.353; HR 1.777; 95% CI 0.528–5.984). The percentage of NK cells was found to predict OS (*P* = 0.005; HR 0.067; 95% CI 0.010–0.435), but not PFS (*P* = 0.659; HR 0.705; 95% CI 0.149–3.340).

**Table 3:** Overall survival and progression-free survival in multivariate Cox analysis in follicular lymphoma

Influencing factors	OS			PFS		
	HR	95%CI	*P*	HR	95%CI	*P*
Pathological grade 3B + composite	0.067	0.010–0.435	0.005	2.933	1.080–7.963	0.035
NK cells < 8%	1.777	0.528–5.984	0.353	0.705	0.149–3.340	0.659

## Discussion

FL is an indolent NHL, and advanced stage FL is considered incurable. However, the prognostic models of FL were also developed based on data from Western countries. Data for Chinese patients are Limited due to the relatively low incidence of FL in China. Meanwhile, because FL patients have a relatively long survival, real-word studies about long-term follow-up of FL patients are also important. Studies have shown that the incidence of FL is correlated with race and region, and the median age of onset was 62.1 years for Caucasians, 57.3 years for Hispanics, 60.7 years for yellow, and 56.8 years for black [12]. The median age of FL patients was 63 years old in our cohorts, and 85 (72%) patients were aged ≥ 60 years. There were 65 (55.1%) male patients, 55 (46.6%) patients with bone marrow involvement, 93 (78.8%) patients with Ann Arbor stage III-IV, 40 (33.9%) patients with grade 3B and composite, 61 (51.7%) patients with FLIPI-2 score ≥ 3, 71 (60.2%) patients with the high risk of PRIMA-PI.

In this cohort, the 3-year OS rate was 82.2%, and the 3-year PFS rate was 57.7%. The pathological grade was found to predict PFS (*P* = 0.035; HR 2.933; 95% CI 1.080–7.963), and the percentage of NK cells was found to predict OS (*P* = 0.005; HR 0.067; 95% CI 0.010–0.435) by multivariate Cox regression analysis. The most common prognostic indices in current use, including the Follicular Lymphoma International Prognostic Index (FLIPI) [13] and PRIMA-Prognostic Index (PRIMA-PI) [14]. In this study, we found that FLIPI2 and PRIMA-PI had the prognostic value for PFS and OS by univariable log-rank test, but were not able to predict PFS and OS upon multivariate Cox regression analysis. The FLIPI was developed in retrospective research to predict OS, while the FLIPI2 was developed to predict PFS as the primary effectiveness objective for model development [5, 15]. Alig et al. compared FLIPI, FLIPI2 and PRIMA-PI the three prognostic assessment models. Among the 475 enrolled patients, FLIPI had the highest sensitivity and PRIMA-PI had the highest specificity in identifying elderly high-risk group patients [16]. In addition, one study had shown that PRIMA-PI had prognostic significance for both time-to-treatment failure (TTF) (*P* = 0.003) and overall survival (OS) (*P* < 0.001), and some patients in the high-risk group had shorter TTF and OS than those in the low-risk group (*P* = 0.001, *P* < 0.001) [17].

In our cohort, the CR rate was 60.2%, which is lower than the 74–85% reported in previous Chinese clinical studies. This discrepancy may be attributed to differences in patient characteristics. Our real-world cohort included a higher proportion of patients with advanced Ann Arbor stage (III-IV, 78.8%), grade 3B or composite histology (33.9%), and high-risk PRIMA-PI scores (60.2%), as well as older patients (72% aged ≥ 60 years). These factors reflect the inclusion of less selected patients compared to clinical trial populations, and may explain the relatively lower treatment response rate.

FL is classified into three distinct pathologic grades (i.e., FL1-3) according to the number of centroblasts per high-power field [2]. FL3 can further be subdivided into FL3a and FL3b in the current WHO classification. FL3b is clinically and biologically more like diffuse large B cell lymphoma and is treated as such. In this study, we found that the pathological grade was found to predict PFS (*P* = 0.035; HR 2.933; 95% CI 1.080–7.963), but not OS (*P* = 0.353; HR 1.777; 95% CI 0.528–5.984) by multivariate cox regression analysis.

The cells of FL interact with a variety of immune cells, including follicular helper T cells (TFH), regulatory cells (Treg), dendritic cells (DC) and tissue cells, etc., which all constitute the tumor microenvironment. Tumor microenvironment is closely related to the occurrence and development of FL with important prognostic value. T lymphocyte subsets including CD3^+^, CD4^+^, and CD8^+^ are the major components of the cellular immune system playing a leading role in antitumor immunity. Identifying the number of CD4^+^ T cells, CD8^+^ T cells, and the CD4^+^/CD8^+^ ratio in peripheral blood can thus represent the immunological state of patients with malignant tumors, and it may also be useful in predicting the prognosis of FL patients. The prognostic value of peripheral blood T-lymphocytes has also been reported in patients suffering from various subtypes of lymphoma [18–21]. The prognostic role of T lymphocyte subsets in diffuse large B-cell lymphoma has been previously reported, but relevant studies are relatively rare in patients with FL. He et al. compared the relationship between peripheral blood CD4^+^ T cell level and prognosis in 127 patients with FL showed that the group with decreased CD4^+^ T cell level was associated with poor PFS and OS. It is suggested that low level of CD4^+^ T cells is an independent risk factor for the prognosis of FL patients [22]. We found that CD4^+^T cells levels < 30% group had poor 3-year OS rates (*P* = 0.001) and 3-year PFS rates (*P* < 0.001) by univariable log-rank test. However, CD4^+^T cells levels was not found to predict PFS and OS by multivariate cox regression analysis. Considering the small sample size and limited research time, it is recommended to continue collecting cases for further study.

Natural killer (NK) cells are important components of the innate immune system with important roles in eliminating viruses, regulating dendritic cells and killing malignant tumor cells [23]. NK cell count is a surrogate marker of host immune status. Previously, Plonquet et al. reported that the association between circulating NK cell number and clinical outcome in DLBCL, a low number of circulating NK cells with a poor response to treatment [24]. In our study, the 3-year OS rates for the NK cells levels < 8% group and ≥ 8% group were 69.5% and 95.3%, respectively (*P* < 0.001) and 3-year PFS rates for the NK cells levels < 8% group and ≥ 8% group were 35.6% and 77.8%, respectively (*P* < 0.001) by univariable log-rank test. However, the percentage of NK cells was found to predict OS (*P* = 0.005; HR 0.067; 95% CI 0.010–0.435), but not PFS (*P* = 0.659; HR 0.705; 95% CI 0.149–3.340) by multivariate cox regression analysis. As we know, FL cells express high levels of HLA-class I [25], which may protect themselves from being recognized by NK cells [26]. Therefore, there is a possibility that NK cells would have a role in the antitumor efficacy of HLA-class I-positive malignancies including FL [25], in accordance with the result of low NK cell levels correlating with inferior prognosis in FL.

Although NK cell percentage was a significant predictor of OS, it did not independently predict PFS in our multivariate model. One possible explanation is that lower NK cell levels may not directly influence the time to disease progression, but instead reflect impaired immune surveillance and host vulnerability to infections or treatment-related complications that contribute to overall mortality. Additionally, the limited number of events and potential competing risks may have reduced the statistical power to detect an effect on PFS.

CD5 is a monomeric type 1 transmembrane glycoprotein with a molecular mass of 67 kDa. Bearing both the extracellular and intracellular domains, CD5 can be treated as a signal transduction molecule [27]. CD5 is normally expressed on T cells and it is also expressed in a small subset of mature B-lymphocytes at low levels [28]. Given the DLBCL patients the front-line chemotherapy R-CHOP (rituximab plus cyclophosphamide, doxorubicin, vincristine, and prednisone), compared to CD5^−^ DLBCL, CD5^+^ DLBCL has an inferior survival rate, with a 5-year overall survival (OS) rate of only 35.5% [29]. Yamaguchi M et al. reported that CD5^+^ DLBCL has more invasive clinical features and is more prone to central nervous system recurrence, extranodal involvement, and with a worse prognosis. So CD5^+^ has been demonstrated to be an independent prognostic factor for DLBCL [30]. Furthermore, CD5 has different clinicopathologic features in different B-cell lymphomas and with different prognosis. For example, it has been reported that patients with CD5^+^ mantle cell lymphoma have poor prognosis [31]. CD5-positive follicular lymphoma (FL), although rare, has been described in a number of case reports. The overall survival (OS) curve of CD5-positive FL was significantly worse than that of CD5-negative cases (*P* = 0.0266), although progression-free survival curves did not show a significant difference (*P* = 0.7899). Moreover, CD5 expression was shown to be an independent poor prognostic factor for OS in both univariate analysis [Hazard Ratio (HR), 3.63; *P* = 0.0464] and multivariate analysis (HR, 57.16; *P* = 0.0001) [32]. Furthermore, Yu Li et al. concluded that CD5 expression in follicular lymphoma is associated with a higher International Prognostic Index, higher rate of transformation to diffuse large B-cell lymphoma, and shorter progression-free survival [33]. In our study, the 3-year OS rates for CD5 positive group and negative group were 71.9% and 93.4%, respectively (*P* < 0.001) and 3-year PFS rates for CD5 positive group and negative group were 34.3% and 81.6%, respectively (*P* < 0.001) by univariable log-rank test. But we did not found CD5 was associated with inferior PFS or OS in multivariable analysis.

CD10 is a cell surface zinc-dependent metalloendopeptidase expressed in normal and neoplastic hematopoietic and non-hematopoietic tissue [34, 35]. CD10 is considered as one of the molecular markers of germinal center B cells. Therefore, CD10 constitutes a canonical element of current subclassification and prognostic algorithms in diffuse large B cell lymphoma (DLBCL), where its expression generally predicts more favorable outcomes [36]. However, CD10 cannot be used alone for independent prognostication of low-grade B cell lymphoma. In our study, in CD10, the 3-year OS rates for positive group and negative group were 94% and 69.9%, respectively (*P* < 0.001) and 3-year PFS rates for positive group and negative group were 77% and 35.7%, respectively (*P* < 0.001) by univariable log-rank test. CD10 negative group had inferior prognosis. But we did not found CD10 was associated with inferior PFS or OS in multivariable analysis.

MUM1(multiple myeloma oncogene-1) also known as IRF4(interferon regulatory factor 4) is a member of the interferon regulatory factor family and has been suggested as a potential therapeutic target in Peripheral T-cell lymphoma (PTCL) due to its expression in T-cell lymphoma [37, 38]. MUM1/IRF4 has been shown to have a crucial role in lymphoid malignancy and is a well-known biomarker of activated B-cell like diffuse large B-cell lymphoma [39]. Mi Hwa Heo et al. reported that MUM1/IRF4 expression was associated with poor survival outcomes in PTCL [40]. Our results showed that in MUM1, the 3-year OS rates for positive group and negative group were 73.3% and 94.5%, respectively (*P* = 0.001) and 3-year PFS rates for positive group and negative group were 41.5% and 79.4%, respectively (*P* = 0.001) by univariable log-rank test, implying that this gene is a potential therapeutic target.

Although the prognostic significance of pathological grade and NK cell levels has been reported in previous studies, our work is the first to validate these findings in a large Chinese real-world population. This distinction is important, as real-world data offer broader representation of clinical diversity and can complement evidence from clinical trials.

This study has several Limitations that should be acknowledged. First, the relatively short follow-up period Limits our ability to evaluate long-term endpoints such as progression of disease within 24 months (POD24), and thus the survival results presented should be interpreted as preliminary. Second, although we adjusted for treatment groups in the multivariate Cox analysis, the heterogeneity in frontline therapeutic regimens (e.g., R-CHOP, BR, GB) and the lack of uniform treatment standards may still have introduced residual confounding. Future studies with longer follow-up and more standardized treatment protocols are needed to confirm these findings.

In conclusion, the FL patients for the score of FLIPI-2 ≥ 3, high-risk (HR) group of PRIMA-PI, the clinical pathological grade of 3B and composite group, with B symptoms, HB < 120 g/L, CD4^+^T cells levels < 30% group, NK cells levels < 8% group, CD5 positive group, CD10 negative group and MUM1 positive group have inferior prognosis of PFS and OS. Among which, the clinical pathological grade and the percentage of NK cells are the independent risk factors for PFS and OS. The baseline peripheral blood NK cell count obtained at diagnosis may represent as an effective biomarker in clinical practice for host immune homeostasis and the tumor microenvironment in FL. Furthermore, this could become the foundation for development of novel therapeutic agents targeting the activation of NK cells. These results support the potential interest of a treatment targeting the activation of NK cells, in particular, in new therapeutic designs including rituximab and obinutuzumab.

## Data Availability

No datasets were generated or analysed during the current study.
